# Insights on Capacitive Interdigitated Electrodes Coated with MOF Thin Films: Humidity and VOCs Sensing as a Case Study

**DOI:** 10.3390/s150818153

**Published:** 2015-07-24

**Authors:** Christos Sapsanis, Hesham Omran, Valeriya Chernikova, Osama Shekhah, Youssef Belmabkhout, Ulrich Buttner, Mohamed Eddaoudi, Khaled N. Salama

**Affiliations:** 1 Sensors Lab, Electrical Engineering Program, King Abdullah University of Science and Technology (KAUST), Thuwal 23955-6900, Saudi Arabia; E-Mails: christos.sapsanis@kaust.edu.sa (C.S.); ulrich.buttner@kaust.edu.sa (U.B.); khaled.salama@kaust.edu.sa (K.N.S.); 2 Advanced Membranes & Porous Materials Center, Chemical Science Program, King Abdullah University of Science and Technology (KAUST), Thuwal 23955-6900, Saudi Arabia; E-Mails: valeriya.chernikova@kaust.edu.sa (V.C.); osama.shekhah@kaust.edu.sa (O.S.); youssef.belmabkhout@kaust.edu.sa (Y.B.); mohamed.eddaoudi@kaust.edu.sa (M.E.)

**Keywords:** metal organic frameworks (MOFs), thin films, porous materials, interdigitated electrodes (IDEs), gas sensor test setup, humidity sensors, capacitive sensors, volatile organic compounds (VOCs)

## Abstract

A prototypical metal-organic framework (MOF), a 2D periodic porous structure based on the assembly of copper ions and benzene dicarboxylate (bdc) ligands (Cu(bdc)·xH_2_O), was grown successfully as a thin film on interdigitated electrodes (IDEs). IDEs have been used for achieving planar CMOS-compatible low-cost capacitive sensing structures for the detection of humidity and volatile organic compounds (VOCs). Accordingly, the resultant IDEs coated with the Cu(bdc)·xH_2_O thin film was evaluated, for the first time, as a capacitive sensor for gas sensing applications. A fully automated setup, using LabVIEW interfaces to experiment conduction and data acquisition, was developed in order to measure the associated gas sensing performance.

## 1. Introduction

Gas sensors are a subclass of chemical sensors that became an emerging research field in recent decades with an expanding behavior in their market. Their working principle is based on the electrical variation introduced in the sensing films by the diffusion or chemical reaction of various gases and vapors in the sensing area. Gas sensors are used in many applications including detecting toxic and flammable gases [1] and food quality control [2]; they are also considered to have potential for use in noninvasive biomedical applications [3,4]. 

Although the need for gas sensors is not new, low-cost and low-power sensing platforms that are effective for long-term routine operation remain beyond the latest research developments. Although gas sensing technology exists in the marketplace, its typical characteristics (bulky, power hungry, complicated instrumental methods and high cost) prevent their mass use. Colorimetric chemical sensors have been proposed for selective gas sensing at low cost [5], however, colorimetric sensors cannot be easily integrated with electronics in a miniaturized microsystem, when compared to other sensing mechanisms, e.g., capacitive sensing. Thus, the development of miniaturized sensors that can be easily integrated with electronics offers prospective to access the needed low-power sensing platforms like lab-on-chip applications.

Metal organic frameworks (MOFs) are crystalline porous materials, which are composed of both organic and inorganic components in a rigid periodic networked structure [6]. The modular nature of MOFs allows the fine tuning of their associated pore size, shape and exposed inner chemical functionality [7,8,9,10]. This unique tunability, not readily accessible in conventional porous materials such as the purely inorganic zeolites, offers great potential for their effective integration and exploration in various sensing applications [7]. Recent reviews show that the selectivity, dynamic range, and integration level of available gas sensors fall short of the requirements of present day applications [9,10]. In response, new materials and devices, such as graphene, carbon nanotubes, and MOFs are being investigated as potential candidates [10,11]. The versatility of MOFs comes from their adjustable pore size, their chemical functionality and their exceptionally high surface area, making these materials attractive for gas sensing applications [10].

A literature review on the use of MOFs for sensing shows that HKUST-1 MOF was used for sensing of volatile organic compounds (VOCs) using gravimetric quartz resonators [12]. Work function based aldehyde detection using Cu-BTC MOF was also reported [13]. In addition, Luminescence characteristics of Y-doped MOF-5 was used for isobutanol sensing [14]. However, these techniques are not suitable for low-power low-cost lab-on-chip applications where the sensor element is integrated with electronics on the same chip. The use of impedance sensing for gas detection with MOFs was proposed [15], but the experimental measurements were done at elevated temperatures (120 °C–240 °C) which is not suitable for low-power portable sensing platforms.

In this paper, the capacitive sensing properties of MOF thin film are studied and evaluated. Capacitive sensors were selected because of their simple structure, compatibility with standard CMOS technology and their ability to operate normally at room temperature assisting low-power applications. In addition, capacitive sensors enable miniaturization reliably and inexpensively. An interdigitated capacitive electrodes (IDEs) structure is used to sense the change in sensing film permittivity upon gas adsorption. We investigate the humidity and VOCs sensing properties of Cu(bdc)·xH_2_O (bdc-benzene-1,4-dicarboxylic acid) MOF thin film on capacitive IDEs. Cu(bdc)·xH_2_O is exploited for its thermal and humidity stable properties [16] and, due to its biocompatible nature, as stimulus responsive antifouling coatings [17]. This type of MOF is used for the first time in gas sensing applications.

Gas sensor testing also needs to be addressed in order to achieve stable and reliable results. Testing consumes valuable time and requires manual effort, which serves as our motivation here to develop, using LabVIEW, a fully automated measurement system. The proposed testing system exceeds the testing capabilities of the current approaches [18,19,20,21] by enabling testing of a wide range of materials for several gases in an optimum and automated process. The setup has paths for water vapor, volatile organic compounds (VOCs) vapors and toxic gases, which can be operated in a single vapor/gas mode or as a mixture. Gas concentration is regulated by adjusting the flow rate through mass flow controllers (MFCs) and the temperature of a chiller bath. Moreover, a hot plate is used to allow operating the testing cell in different temperature levels. Altering the temperature expands the range of materials that can be tested because some materials require an elevated temperature to achieve high performance. A gas bubbler was used as a vapor dosing system because it provides a steady and controllable flux, which is important for measurements accuracy. We also tested polyimide as a sensing film because of its known linear behavior in humidity applications [22] to verify the stability of the measurement setup.

## 2. Sensing Element

### 2.1. Interdigitated Electrodes (IDEs)

Interdigitated electrodes (IDEs) were fabricated in the KAUST Nanofab on 4-inch (100 mm) <100> p-type silicon wafers of 2.5–4.0 Ω·cm electrical resistivity from MEMC. A 2 μm oxide layer was thermally grown for electrical isolation. Silicon wafers and 2 μm oxide layer were selected to imitate IDEs grown in a standard CMOS process. The IDEs were patterned using a subtractive process, which gives better results compared to lift-off, given the thin and long IDEs fingers. 

First, a stack of 10 nm Ti and 300 nm Au was deposited via physical vapor deposition (PVD) using 400 W DC power for 60 s and 400 W DC power for 360 s respectively in an ESC reactive and metal sputter system. Titanium (Ti) was used as an adhesion layer rather than Chromium (Cr) because it is more easily dry etched. Next, optical lithography was used to pattern the electrodes. An image reversal photoresist (AZ5124E) was spun at 3000 rpm for 30 s yielding a 1.6 μm photoresist layer, followed by soft baking at 105 °C for 120 s. A 5 inch dark-field optical mask was created using a Heidelberg uPG101 Laser Mask Writer. The photoresist was patterned using contact lithography and a 90 mJ/cm^2^ broadband exposure dose. For image reversal, the wafer was then heated again at 120 °C for 120 s followed by flood exposure. Puddle developing for 60 s using AZ726 was performed in SUSS MicroTec Delta 12AQ spray develop system. 

**Figure 1:**
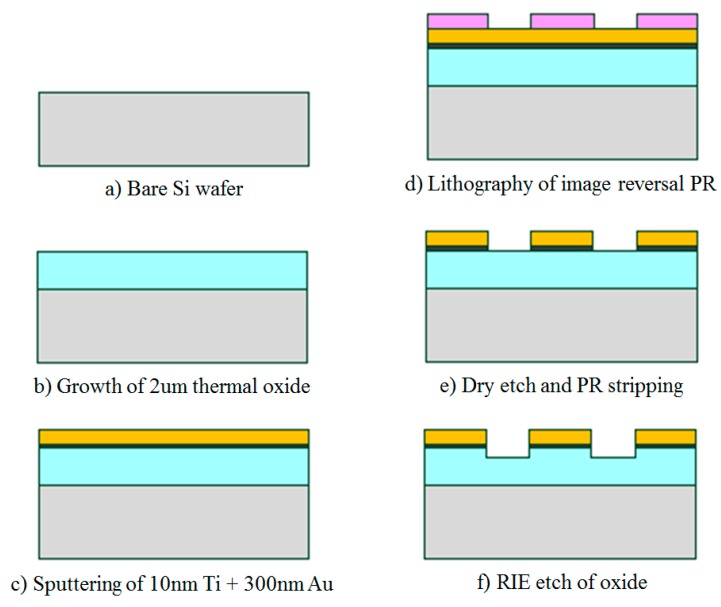
Interdigitated electrodes (IDEs) fabrication process.

**Figure 2:**
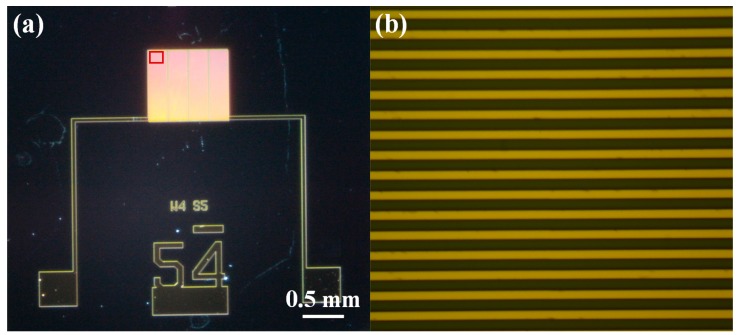
(**a**) Optical microscope image of the fabricated IDE device; (**b**) enlarged image of enclosed area in (**a**).

The metal layer was patterned by dry etching using the Oxford Instruments PlasmaLab System. A 30 mL/min flow of Ar was used for 4 min with 150 W RF power and 1000 W ICP power. The exposed oxide thickness was further verified using Nanospec 6100 Reflectometer to ensure that the metal layer was properly etched. Dry etching prevented the acetone from completely stripping the photoresist layer and thus O_2_ plasma had to be used. The O_2_ plasma step also partially etches the oxide between the IDEs’ fingers. This enhances the sensitivity because the thin film completely fills the region between the fingers, which has the maximum electric field density. The process of fabrication and the fabricated IDEs are depicted in Figure 1 and Figure 2, respectively. The IDEs were designed with 4 μm fingers and 5 μm spaces due to the limitations of the lithography tools. Two Au wires and contact pads were patterned to perform the electrical measurements.

### 2.2. Cu(bdc)·xH_2_O MOFs Thin Film

**Figure 3:**
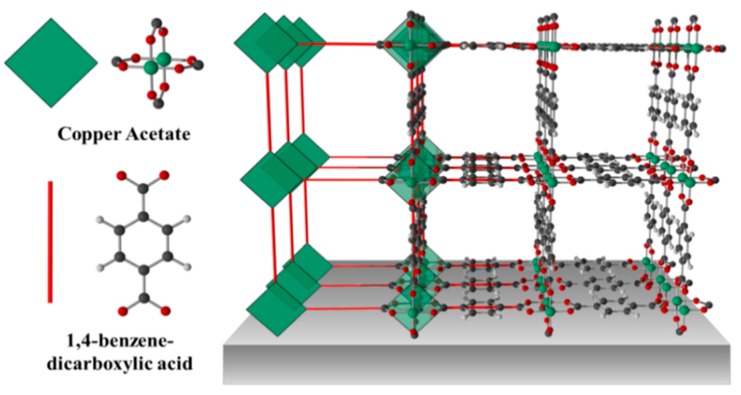
Schematic representation of the 2D Cu(bdc)·xH_2_O MOF thin film structure on a sensor chip.

**Figure 4:**
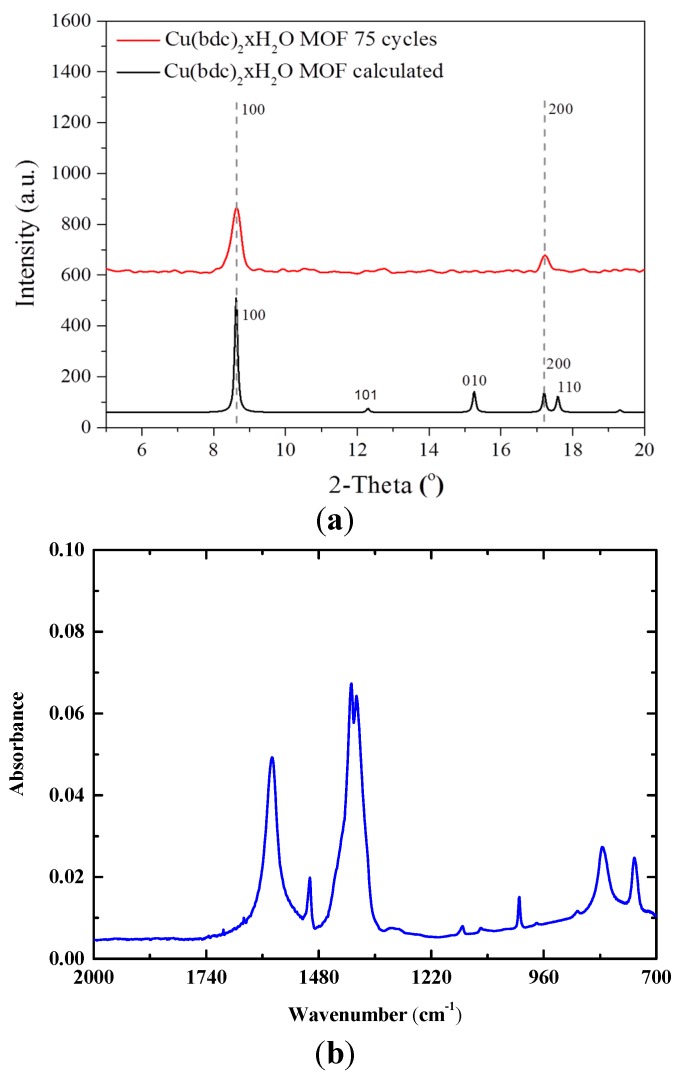
(**a**) XRPD of Cu(BDC)·xH_2_O MOF thin film grown on a sensor chip (red) and its calculated pattern (black); (**b**) IR spectrum of the Cu(BDC)·xH_2_O MOF thin film.

**Figure 5:**
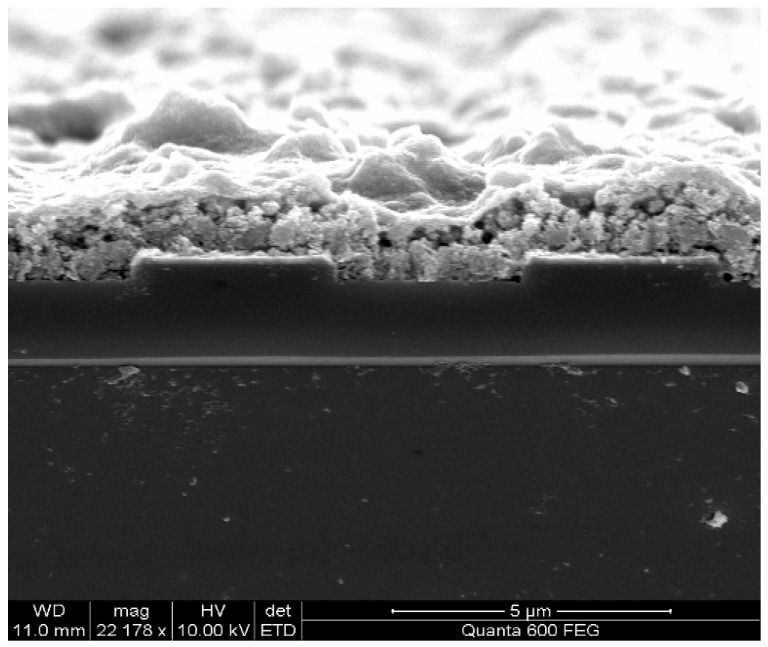
SEM of Cu(bdc)·xH_2_O film.

The growth of a Cu(bdc)·xH_2_O thin film using the liquid-phase epitaxy method has previously been described in detail [16]. In brief, the sensor substrate is first modified with COOH-terminated self-assembled monolayers (SAM) by immersing the bare substrate for 24 h in ethanolic solution of 16-mercaptohexadecanoic acid [23,24,25], then the Cu(bdc)·xH_2_O thin film was prepared by alternatively immersing of the modified sensor substrate into ethanolic solutions of the building components: copper acetates and H_2_bdc (bdc-benzene-1,4-dicarboxylic acid), and rinsing with pure ethanol in between. Figure 3 illustrates the chemical structure. The thickness of the thin film was controlled by the number of growth cycles. The formation of MOF thin film was confirmed using both powder X-ray diffraction (PXRD) and IR spectrum (Figure 4), which shows that the 75 cycles crystalline and highly oriented thin film was obtained. The SEM image in Figure 5 shows that thin films are highly homogenous with an overall thickness of about 1.5 µm.

## 3. Gas Sensing Setup

### 3.1. Measurement System Implementation

**Figure 6:**
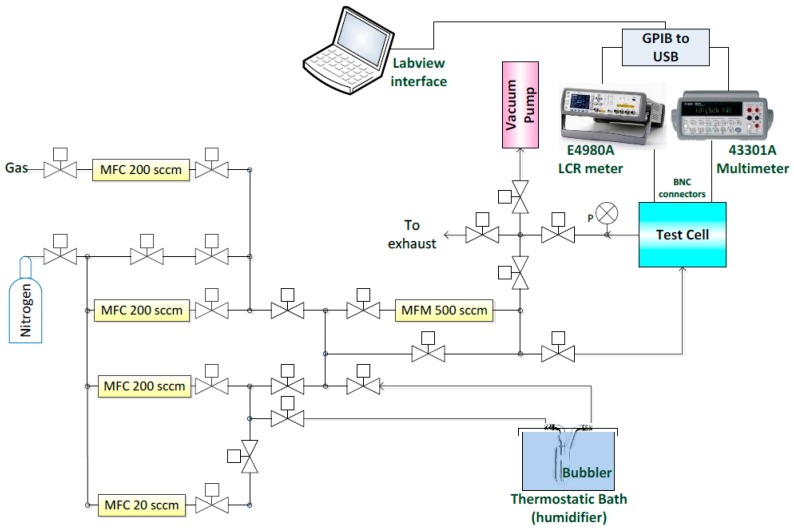
Configuration of the testing setup.

The system was designed to be a complete gas setup for testing the effect of humidity, VOC vapors and toxic gases on sensitive films. Figure 6 illustrates the four basic supply paths in the setup controlled by MFCs for:

- diluted VOC or toxic gas cylinders,
- dry N_2_ for diluting other gases/vapors in mixtures,
- carrier gas to generate water vapor, and
- carrier gas to generate VOC vapor.

MFCs, provided by Alicat Scientific Inc. (Tucson, AZ, USA), were used for regulating the mass flow in the above mentioned paths. The maximum allowed flow per MFC in mL/min and its usage are described in Table 1. A second path of dry N_2_ is used for purging with the flow to be regulated by a Vernier metering valve (Swagelok). Stainless steel delivery lines were used on the setup with the exception of the vapor path, which was connected to the gas bubbler with special perfluoroalkoxy alkane (PFA) tubing allowing flexibility and resistance to the VOCs vapors.

**Table 1:** MFC usage and maximum values.

MFC	Usage	Maximum Value (mL/min)
A	VOCs carrier	20
B	H_2_O carrier	200
C	Dry N_2_ carrier	200
D	Diluted gas cylinder	200

Paths for generating water and VOC vapors were routed to the bubbler, which was exposed to carrier flow rates up to 200 mL/min for humidity testing and up to 20 mL/min for VOCs. The carrier flow passes via the bubbler, which is held at a fixed temperature using a cooling system (Chiller F12-MA) by Julabo GmbH. The vapor is generated through the flow of N_2_ inside the bubbler creating an air-water (or VOC) mixture. This is based on the difference in temperature of the testing element (ambient) and the lower temperature that should be provided by the chiller. The ratio of the partial pressure of water vapor in the mixture to the saturated vapor pressure of water at ambient temperature is the relative humidity (RH%). Pressure values can be calculated through Antoine’s Equation (1):

(1) $$log_{10}P=A-\frac{B}{C+T}$$

where P is the pressure, T is the temperature, A, B and C are parameters that are specific per material in a constrained range of temperatures.

The generated vapor was further diluted by mixing it with dry N_2_. This mixture then reached the testing chamber passing through a mass flow meter (MFM), which measures the overall mass flow. 

**Figure 7:**
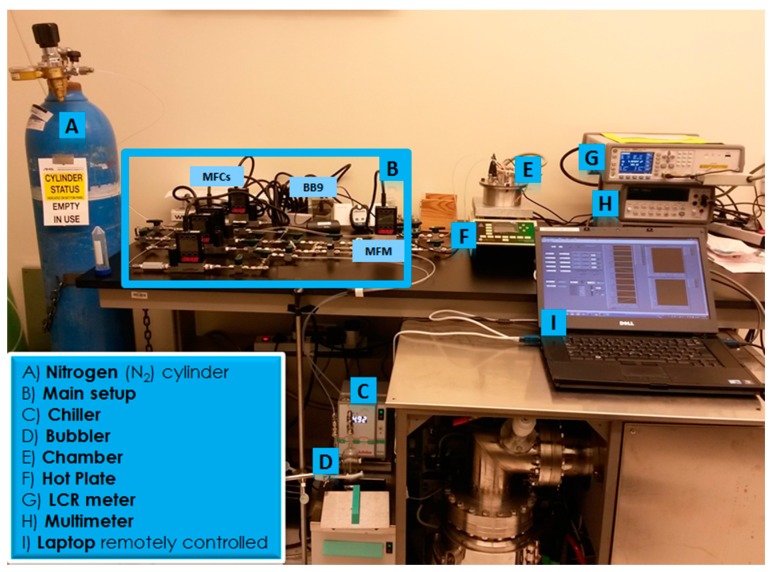
Implemented testing setup.

A custom stainless steel testing chamber was equipped with a gas inlet (1/4″ FNPT), gas outlet (1/4″ FNPT) and four hermetic BNC electrical connectors. For quality purposes, the cell passed a leakage test under high pressure and under vacuum using a turbo-molecular pump. The volume of the cell was approximately 400 cm3 $\;\left(\sim \text{π}\times 5{\text{cm}}^{2}\times 5\text{cm}\right)$. The chamber encompassed two sensors: a commercial humidity sensor (Honeywell HIH-4000-003) and the implemented capacitive sensor IDEs. The commercial sensor indicates the humidity level inside the chamber, which allows the detection of a difference in capacitance for the implemented sensor at different humidity levels. The output voltage of the commercial sensor was measured by a multimeter (Agilent 43301A). An LCR meter (Agilent E4980A) measures the capacitance of the sensors under test. The outlet of the chamber goes to the exhaust system or can be directed to a vacuum pump (Mastercool Inc., Randolph, NJ, USA). The test chamber is positioned on a hot plate (Torrey Pines Scientific EcoTherm HS60) for possible testing at elevated temperatures. The implemented measuring testing setup is depicted in Figure 7.

### 3.2. Experiment Automation

The MFCs, the chiller and the hot plate communicated with the PC via RS232 communication using RS232 to USB cables. All experiments were automated for setup’s usage simplicity, while manual control was also allowed. For guaranteed safe operation, the pressure at each MFC was continuously monitored by the LabVIEW interface throughout the operation. If the pressure exceeded a certain threshold, the MFCs were automatically turned off. Data acquisition by controlling the LCR meter and the multimeter was also automated in NI LabVIEW.

## 4. Results and Discussion

### 4.1. Humidity Experiments

**Figure 8:**
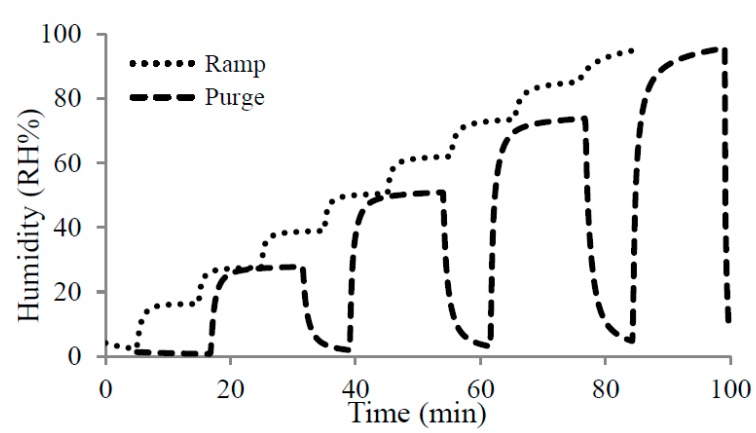
Ramp and purge experiment for the commercial sensor.

For the humidity testing of the sensor, two different experimental approaches were conducted. The first, purge experiment, required purging in between the main steps. The relative humidity level was varied by changing the carrier flow of the bubbler’s inlet and dilution of dry N_2_ from 0 to 200 mL/min and from 200 to 0 mL/min by steps of 50 mL/min respectively. The second experiment was an increasing ramp of relative humidity in the absence of purging. The carrier and dilution flow ranged from 0 to 200 mL/min and from 200 to 0 mL/min by steps of 25 mL/min respectively. Figure 8 presents the results of each experiment for the commercial sensor. 

The purge experiment was repeated three times to prove the reproducibility of the results. The time to reach a saturated value depended on the material and the experiment. Cu(bdc)·xH_2_O needed 10 min and 15 min per step for ramp and purge experiments, respectively. The temperature of the chiller bath was maintained at 17 °C while the ambient temperature was measured at 20 °C. We found similar results from both ramp and purge experiments. The ramp experiment provided measurements for more humidity levels, and for this reason, was used in the following figures. All capacitances were measured at 1 MHz with an LCR meter (E4980a). 

**Figure 9:**
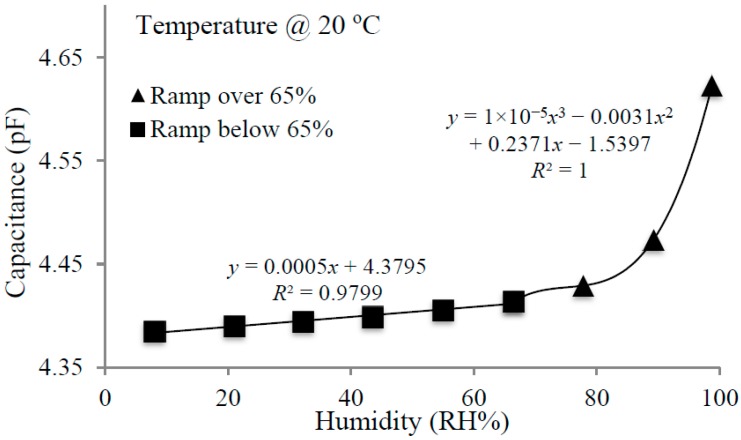
Capacitance *vs.* humidity plot using Cu(bdc)·xH_2_O MOF.

Cu(bdc)·xH_2_O is a porous MOF material having open metal sites that could be occupied or free. This MOF has efficient adsorption and storage of analytes, and thus, should by nature have a high sensitivity for gas and vapor adsorption, driven mainly by pore filling. The water vapor can permeate inside the film and the size of the pores will determine the amount of vapor that can be stored. Figure 9 shows that the humidity behavior of the MOF film is linear until a relative humidity of 65%, following equation *y* = 0.0005*x* + 4.3795, with a linear fit of *R*² = 0.9799. Over 65%, the curve follows a third order polynomial behavior; this change to non-linearity can be explained by the hydrogen bonding interactions of the water vapor with water coordinated to the open metal sites and the exponential characteristic course of the water vapor saturation concentration. 

**Figure 10:**
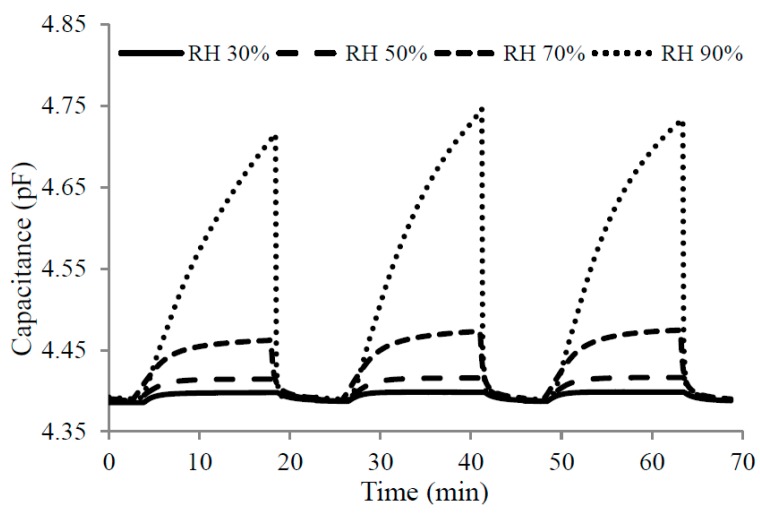
Reproducible Results for the purging experiment for Cu(bdc)·xH_2_O Film.

Humidity levels above 65% have a considerable effect on the sensitivity of Cu(bdc)·xH_2_O. Because of its porous nature, a greater amount of vapor is able to adsorb on the MOF thin film. While the vapor storage ability of this MOF has been proven above 65%, the response time is very low. As an indication of reproducibility, three consecutive measurements are shown in Figure 10. 

The response and recovery times could not be accurately evaluated because the system has a maximum flow rate of 200 mL/min and thus the chamber requires a relatively long time (3–4 min) to reach from 10% to 90% RH. Nevertheless, we estimated sensor’s response time by comparing it to the commercial one. Cu(bdc)·xH_2_O reacts to humidity change similarly to the commercial following its response. 

Minor differences in the results of repeated experiments are mostly due to slight variations in RH levels for each run of the experiment, especially in the last step, where the humidity level was more elevated; even a small difference can have a great impact on the capacitance because the response to humidity is exponential.

### 4.2. Volatile Organic Compounds Experiments

We used a gas bubbler filled with the VOC in its liquid phase to generate its vapor. Dry nitrogen was used as a carrier gas to transfer the generated vapor to the outlet. This design, along with a steady flow of MFCs provided a continuous flow of vapor generation. The calculation of the output flow was based on the following equation [26]:

*F_out_* = (*a* +1)*F_c_* (2)

where $F_{out}$ is the output flow, $F_{c}$ is the carrier flow, and a is the ratio of the saturated vapor pressure to the input pressure, which can be calculated through Antoine’s equation for both bath and ambient temperatures.

The generated vapor was diluted with dry nitrogen before insertion into the testing chamber. The final concentration in parts per million (ppm) can be calculated by Equation (3):

(3) $$C_{ppm}=\;\frac{aF_{c}}{F_{d}+\left(a+1\right)F_{c}}\times 10^{6}$$

We tested the VOC analytes acetone, ethanol, methanol and toluene. Wide variations in concentrations were achieved by varying either the bath temperature or/and the flow rate in the bubbler. For the purge experiment for acetone, ethanol and methanol, the flow rate was varied for the carrier from 0 to 10 mL/min and the dilution ($F_{d}$) from 200 to 190 mL/min by a step of 2.5 mL/min. The dilution flow was acting complementarily to the carrier with their summation to reach 200 mL/min in order to achieve a steady flow rate of 200 mL/min in the chamber. The bath temperature for all these experiments was set at 0 °C, while ambient temperature was measured at 22 °C. The time per step was 7 min, while the purging time between the main steps was 3.5 min. 

A gas cylinder was used with diluted toluene in nitrogen at a concentration of 500 ppm. The experiment was based on varying the gas and dilution flow from 0 to 200 mL/min and from 200 to 0 mL/min by steps of 50 mL/min respectively. The experiment time was 7 min per step with a purging time of 3.5 min.

**Figure 11:**
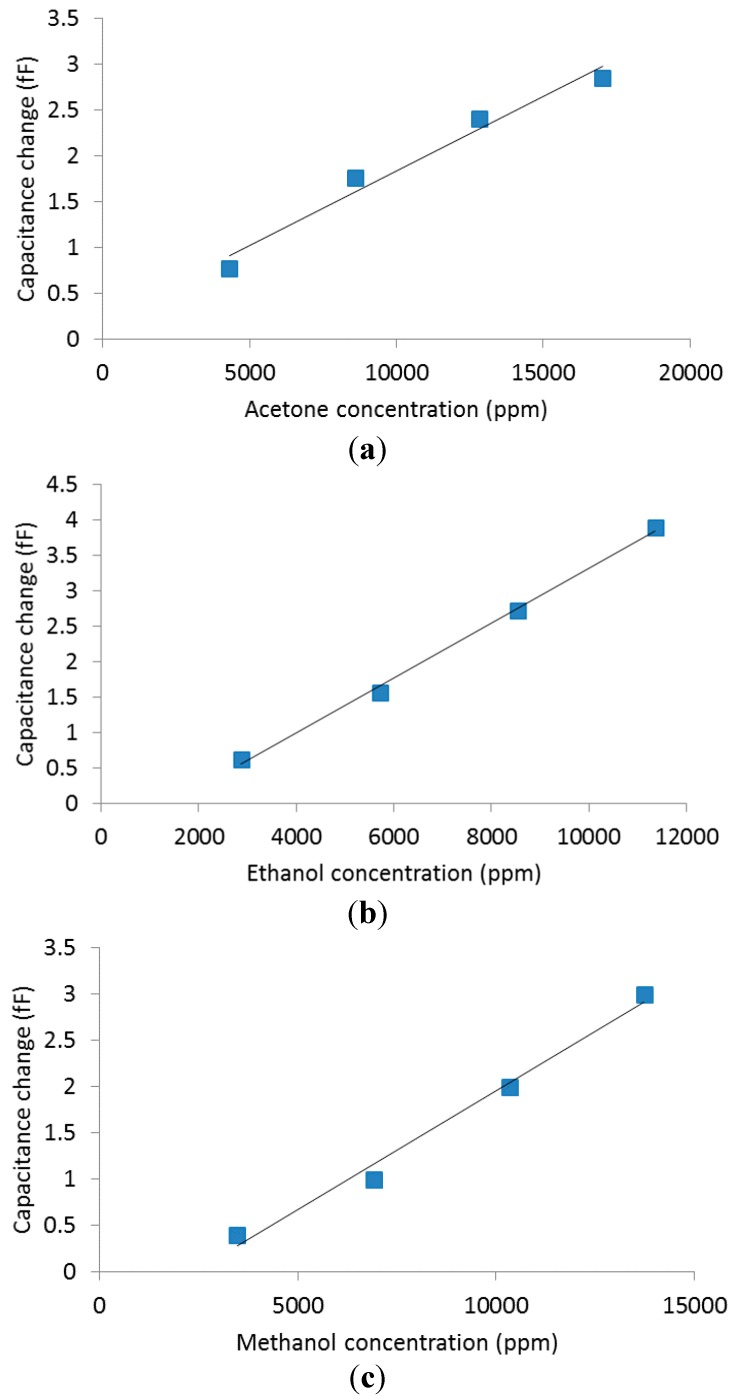

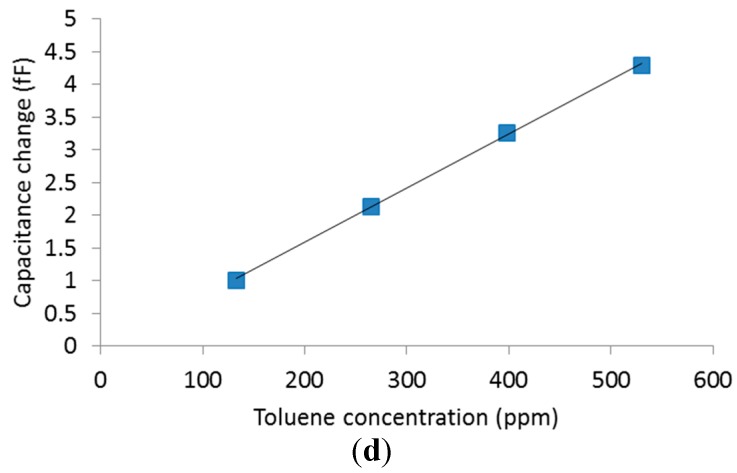
MOF response (change in capacitance) for different concentrations of (**a**) acetone, (**b**) ethanol, (**c**) methanol and (**d**) toluene. The baseline capacitance is approximately 4.4 pF. Measurements were done at a temperature of 22 °C.

Figure 11 shows that the change of capacitance of Cu(bdc)·xH_2_O has linear behavior for acetone, ethanol and methanol. The similarity in the responses by all three vapors is likely due to similarity in their chemical nature and size of their molecules. Moreover, at very low concentrations (125–500 ppm) of Toluene, MOF also responded linearly. Due to the large size of the molecule, the diffusion is slower. The film is more sensitive to Toluene due to the π-π interaction with the benzene rings of the organic part of the MOF [27].

All the experiments performed showed reproducible results and recoverability. The sensors are proven to be reusable since the same samples were reused and cleaned for each experiment by N_2_ purging.

The sensing mechanism is due to the change of the permittivity of the thin film upon analyte adsorption. The clean IDEs are made of gold on top of silicon dioxide layer. The silicon dioxide is not sensitive to any of the studied analytes so there will be no capacitance change. In case of the IDEs modified with the SAM, the SAM layer will not affect the measurement since the thickness of the SAM layer is about 2 nm, which is negligible compared to the MOF thin film thickness (1.5 mm). In addition, control experiments for the growth of 75 cycles of copper acetate alone and the H_2_bdc ligand alone were done and no growth was observed on the IDEs in both cases as was confirmed by XRD and SEM, which confirms that the observed sensing behavior is due to the MOF thin film.

## 5. Conclusions

Gas sensing properties of the Cu(bdc)·xH_2_O MOF thin film is reported. An automated gas sensor test setup is designed and implemented, allowing testing of a wide range of concentrations of different gases and vapors. The MOF thin film is sensitive to humidity and shows an exponential response at elevated humidity levels. The response of the MOF thin film to VOCs is linear.
